# Association of Systemic Inflammation Indices With Mortality in Coronary Atherosclerosis Patients With and Without Standard Modifiable Risk Factors

**DOI:** 10.1155/mi/8830121

**Published:** 2026-01-10

**Authors:** Weiren Yan, Bingqian Zhang, Xiaoyan Zhang, Xinsheng Li, Yao Yu, Yuguo Liu, Lei Guo, Haichen Lv

**Affiliations:** ^1^ Department of Cardiology, The First Affiliated Hospital of Dalian Medical University, Dalian, China, dlmedu.edu.cn; ^2^ Department of Radiology, Fuyang Hospital of Anhui Medical University, Fuyang, China

**Keywords:** coronary heart disease, mortality, standard modifiable risk factors, systemic inflammation

## Abstract

**Background:**

Standard modifiable risk factors (SMuRFs) are important causative factors leading to coronary atherosclerosis. However, a significant number of individuals develop coronary atherosclerosis despite the absence of SMuRFs. Inflammation is another major cause of atherosclerosis, and this study aims to investigate the association of the novel inflammatory markers systemic immune inflammatory index (SII) and systemic inflammatory response index (SIRI) with mortality in patients with coronary heart disease (CHD) with and without SMuRFs.

**Methods:**

In this study, we included 1708 CHD participants from the 1999–2018 National Health and Nutrition Examination Survey (NHANES). Patients were categorized into ≥ 1SMuRF and SMuRF‐less groups by questionnaire and serologic testing. SII and SIRI were categorized into four groups according to quartiles. Multivariate weighted Cox regression was used to explore the risk factors associated with mortality in patients with or without SMuRFs. Restricted cubic spline (RCS) curve was used to assess their nonlinear correlation.

**Results:**

In patients with ≥1 SMuRF, all‐cause mortality (SII:hazard ratio [HR] 1.47, 95% confidence interval [CI] 1.18–1.84, *p*  < 0.001; SIRI:HR 1.66, 95%CI 1.31–2.10, *p*  < 0.001) and cardiovascular mortality (SII:HR 1.52, 95%CI 1.07–2.17, *p* = 0.020; SIRI:HR 1.63, 95%CI 1.11–2.38, *p* = 0.011) were significantly higher in the SII Q4 and SIRI Q4 group compared to the SII Q1 and SIRI Q1 group, respectively. In patients with SMuRF‐less, the incidence of all‐cause mortality was also significantly higher in the group with higher levels of SII, SIRI (SII:HR 3.32, 95%CI 1.45–7.59, *p* = 0.004; SIRI:HR 4.25, 95%CI 1.67–10.80, *p* = 0.002), but no significant difference was observed in cardiovascular mortality for SII (SII:HR 2.21, 95%CI 0.54–8.97, *p* = 0.272), while a significant association was found for SIRI (SIRI:HR 11.69, 95%CI 1.43–95.21, *p* = 0.028). The RCS analysis showed a linear trend between high levels of SII, SIRI, and elevated all‐cause mortality, and cardiovascular mortality in patients with ≥1 SMurRF. In contrast, a positive linear trend between SII, SIRI, and all‐cause mortality, but no significant association with cardiovascular mortality was observed in the group with SMuRF‐less.

**Conclusions:**

The findings showed that SII and SIRI were positively associated with all‐cause mortality in a population with CHD irrespective of the presence or absence of SMuRFs. The present study suggests that inflammation may be an important factor in the poor prognosis of patients with no specific cardiovascular risk factors, which needs to be further argued by more prospective studies.

## 1. Introduction

Coronary heart disease (CHD) is one of the leading causes of death globally, and the high prevalence of CHD is becoming an important public health issue with the accelerating aging process [1, 2]. Diabetes mellitus, hypercholesterolemia, hypertension and, smoking are recognized as modifiable risk factors for CHD, and the prevention and treatment of CHD has improved dramatically with the control and intervention of modifiable risk factors [3, 4]. However, even after controlling these risk factors, global CHD morbidity and mortality remain high [5], suggesting that there are still potentially unknown risk factors that influence the pathogenesis and prognosis of the disease.

In recent years, a series of studies have shown that an increasing number of CHD patients have been found to be free of standard modifiable risk factors (SMuRFs) and are rising at a steady and significant rate [6, 7]. A multicenter study from Australia found that the incidence of myocardial infarction (MI) in patients with SMuRF‐less increased from 14% to 23% between 1999–2017, with a large and increasing proportion of MI cases occurring independently of SMuRFs [6]. This is consistent with findings from a large global meta‐analysis of over 1.2 million acute coronary syndrome (ACS) patients, which reported that approximately 12% of patients were SMuRF‐less [7]. In contrast, patients with SMuRF‐less did not show a better prognosis compared with patients with at least 1 SMuRF (≥ 1 SMuRF), especially in patients with ST–segment elevation MI (STEMI) [8], and although this may be related to the lack of evidence‐based pharmacotherapy in this population, it may also be attributable to currently unidentified risk factors. This paradox highlights a significant clinical management gap: The absence of tailored risk stratification tools designed specifically for SMuRF‐less patients, who may be undertreated because they are perceived as "low risk." Therefore, there is an urgent need to find a new biomarker to effectively identify the prognosis of SMuRF‐less patients.

Chronic inflammation plays an important role in the pathogenesis of coronary atherosclerosis. Even in the absence of traditional risk factors, inflammation itself can drive arterial proliferation and modulate aspects of plaque biology, thereby triggering thrombotic complications of atherosclerosis [9, 10]. Systemic immune inflammatory index (SII) and Systemic inflammatory response index (SIRI), two novel inflammatory markers consisting of platelets and three subtypes of leukocytes, have shown better prediction in tumors such as colorectal and esophageal cancers, compared to the traditional inflammatory markers neutrophil‐to‐lymphocyte ratio (NLR) and platelet‐to‐lymphocyte ratio (PLR) value [2, 11]. In addition, many studies have shown that SII and SIRI are also strongly associated with cardiovascular and all‐cause mortality [12, 13]. While traditional inflammatory markers such as C‐reactive protein (CRP) and the NLR have shown prognostic value, composite indices like SII and SIRI, which integrate counts of multiple immune cell types (neutrophils, platelets, lymphocytes, and monocytes), may offer a more comprehensive reflection of the underlying systemic inflammatory and immune status. However, the correlation between SII and SIRI and mortality in patients with coronary atherosclerosis with and without SMuRFs is unknown. To date, there is a notable lack of research focusing on the prognostic value of inflammatory biomarkers specifically within the SMuRF‐less population. Given the clinical management gap for these patients, who often face a paradoxical high risk of adverse outcomes, there is an urgent need for novel risk stratification tools. Therefore, this study aimed to examine the relationship between SII and SIRI and mortality in coronary atherosclerosis patients with and without SMuRFs, investigating the potential utility of these inflammatory markers to refine prognosis in this special population. Simultaneously, a brief correlation analysis was conducted incorporating the relatively well‐established inflammatory indices NLR and PLR for comparison. By evaluating these systemic inflammation indices in SMuRF‐less and SMuRF ≥1 patients, this study may help refine risk stratification beyond conventional risk factors and identify high‐risk individuals who might otherwise be overlooked.

## 2. Methods

### 2.1. Study Population

**Figure 1:**
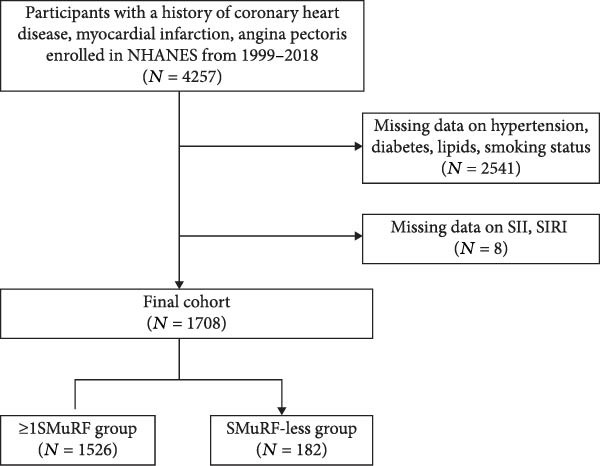
The flow chart.

The National Health and Nutrition Examination Survey (NHANES) database is a population‐based cross‐sectional survey program conducted by the Centers for Disease Control and Prevention (CDC) and the National Center for Health Statistics (NCHS). Detailed demographic information was collected through interview surveys, physical examination and laboratory tests, and followed up over time. All data were made freely available and unrestricted reuse was allowed through an open license (http://www.cdc.gov/nchs/nhanes.htm). All NHANES protocols were approved by the NCHS Ethics Committee (NCHS, 2012) and written informed consent was obtained from all participants. This study included data from 10 continuous cycles of the NHANES database (1999–2018). CHD patients were defined as an individual who answered “yes” to the following question on the NHANES questionnaire: “Has a doctor or other health professional ever told you that you had a heart attack also called myocardial infarction?” “Has a doctor or other health professional ever told you that you had coronary heart disease?” and “Has a doctor or other health professional ever told you that you had angina also called angina pectoris?”. This method of identifying patients with CHD has been used in previous studies [14, 15]. A total of 4257 participants were enrolled in the study, after which 2541 participants with incomplete histories and eight participants lacking calculated SII and SIRI data were excluded, resulting in the inclusion of 1708 CHD patients with complete data. Figure 1 displays the participants selection procedure.

### 2.2. Definition of ≥1 SMuRF and SMuRF‐Less

SMuRF was defined as the presence of at least one of the following: current smoking status, hypercholesterolemia, diabetes, or hypertension [16]. Current smoking status was defined as an individual who answered “every day” or “some days” to the following question: Do you now smoke cigarettes? Hypercholesterolemia was defined as previous or ongoing oral low‐density lipoprotein cholesterol (LDL‐C) lowering therapy with an LDL‐C concentration ≥3.5 mmol/L or a total cholesterol (TC) concentration ≥ 5.5 mmol/L. Diabetes was defined as a self‐reported diagnosis or ongoing use of hypoglycemic medication including insulin or other oral hypoglycemic agents, or a fasting blood glucose (FBG) level ≥ 7 mmol/L or HbA1c ≥ 6.5%. Hypertension was specifically defined as a systolic blood pressure (SBP) of ≥140 mmHg, a diastolic blood pressure (DBP) of ≥90 mmHg, or the current use of antihypertensive medication. SMuRF‐less is defined as the absence of these SMuRFs above.

### 2.3. Definition of SII and SIRI

SII is defined as (platelet counts × neutrophil counts)/lymphocyte counts and SIRI is defined as (neutrophil counts × monocyte counts)/lymphocyte counts [17]. These indices were calculated using laboratory data obtained at the baseline examination only. SII and SIRI were categorized into four groups according to quartiles, and the relationship between these two indices and mortality in patients with CHD with or without SMuRFs was assessed.

### 2.4. Determination of Mortality Outcomes

The NCHS has linked data collected from several NCHS population surveys with death certificate records from the National Death Index (NDI). Publicly available death data through December 31, 2019 (https://www.cdc.gov/nchs/data-linkage/mortality-public.htm) were used for this study. Follow‐up duration for each participant was calculated from the date of the baseline interview to the date of death or the end of the follow‐up period (December 31, 2019), whichever came first. Participants who were still alive at the end of the follow‐up period were censored. Statistics were determined according to the International Statistical Classification of Diseases, 10th edition (ICD‐10): all‐cause deaths, cardiovascular disease deaths (ICD‐10:054‐068), and cancer deaths (ICD‐10:019‐043) [18].

### 2.5. Covariates

This study accounted for a range of covariates, including demographic characteristics (age, education, race, marital status, poverty‐to‐income ratio [PIR], body mass index [BMI], alcohol consumption, and smoking habits), medical history (diabetes and hypertension), laboratory test results (TC, triglycerides [TG], high‐density lipoprotein cholesterol [HDL‐C], alanine aminotransferase [ALT], aspartate aminotransferase [AST], and estimated glomerular filtration rate [eGFR]). Chronic kidney disease (CKD) was defined as an eGFR< 60 mL/min/1.73 m^2^. Hyperlipidemia was defined as any one or more of the following: TC ≥ 6.2 mmol/L, LDL‐C ≥ 4.1 mmol/L, TG ≥ 2.3 mmol/L, and HDL‐C ≤ 1.0 mmol/L.

### 2.6. Statistical Analysis

To account for the complex, multistage probability sampling design of the NHANES survey, all analyses incorporated the appropriate sampling weights, strata, and primary sampling units (PSUs) to generate nationally representative estimates. Continuous variables were presented as mean ± standard deviation (SD), and categorical variables were expressed as counts and percentages, and were compared using the ANOVA and Pearson chi‐square test. Event‐free survival was estimated using Kaplan–Meier survival curves and compared with the log‐rank test. Multivariate Cox regression models were performed to evaluate the relationship between SII, SIRI and mortality. Schoenfeld residual test was used to verify the assumption of proportional hazards in the Cox analysis. Three models were used to assess the relationship: Crude model was unadjusted; Model 1 adjusted for age, race, education, and marital status; Model 2 further adjusted for smoking, alcohol consumption, BMI, hypertension, diabetes, CKD, HDL‐C, TG, TC, LDL‐C, ALT, and AST. Restricted cubic splines (RCSs) analysis was performed to examine potential nonlinearity in the associations and to depict overall trends. RCS analyses with three knots placed at the 10th, 50th, and 90th percentiles of the SII or SIRI distributions were performed to examine potential nonlinearity in the associations and to depict overall trends. Statistical analysis was conducted using R Version 4.1.3, with a *p*‐value < 0.05 (two‐sided) considered statistically significant.

## 3. Results

### 3.1. Baseline Characteristics of Population Stratified by SII/SIRI

A total of 1708 CHD patients were included in this study, including 1526 patients with≥ 1 SMuRF and 182 patients with SMuRF‐less. The mean age of these patients was 67.29 ± 12.1 years, and the majority were male (61.4%) and non‐Hispanic White (59.2%). During a median follow‐up of 6.58 years, 737 patients (43.1%) died, and a total of 259 (15.1%) died of cardiovascular diseases (CVDs). All patients were divided equally into four groups according to quartiles of SII, SIRI, and as shown in Table 1, the number of women, non‐Hispanic Whites, and people with drinking habits increased with increasing SII in patients with ≥1 SMuRFs, however, there was no such pattern in the population with SMuRF‐less. And when grouped in SIRI quartiles, patients with ≥1 SMuRFs in the SIRI Q4 group were older, more likely to be male, had lower levels of LDL‐C and HDL‐C, and higher eGFR compared to the SIRI Q1 group. However, the difference was not found in patients with SMuRF‐less (Table 2). Additionally, we found that SII and SIRI levels increased in conjunction with each other.

**Table 1:** Baseline information according to quartiles of SII.

Variable	≥ 1SMuRF				*p*	SMuRF‐less				*p*
	Q1 (*N* = 382)	Q2 (*N* = 381)	Q3 (*N* = 381)	Q4 (*N* = 382)		Q1 (*N* = 46)	Q2 (*N* = 45)	Q3 (*N* = 45)	Q4 (*N* = 46)	
SII	515.40 ± 98.96	742.29 ± 60.67	970.99 ± 85.93	1644.88 ± 737.18	< 0.001	486.93 ± 90.10	692.72 ± 52.48	884.76 ± 65.48	1441.92 ± 474.21	< 0.001
Age (years)	65.02 ± 12.30	64.49 ± 11.68	64.86 ± 12.21	66.12 ± 13.36	0.661	65.58 ± 10.65	63.17 ± 15.77	63.41± 14.88	64.26 ± 14.40	0.884
Gender (*n* [%])					0.005					0.092
Male	247 (67.4)	242 (62.8)	217 (58.1)	207 (50.4)		38 (77.7)	36 (84.5)	31 (56.7)	31 (69.8)	
Female	135 (32.6)	139 (37.2)	164 (41.9)	175 (49.6)		8 (22.3)	9 (15.5)	14 (43.3)	15 (30.2)	
Race (*n* [%])					0.033					0.2
Mexican American	39 (4.6)	41 (3.0)	57 (4.9)	34 (3.4)		4 (2.1)	4 (6.0)	5 (4.5)	6 (6.5)	
Non‐Hispanic Black	92 (13.2)	74 (9.8)	60 (8.5)	40 (5.3)		3 (2.8)	2 (2.0)	0 (0.0)	3 (5.4)	
Non‐Hispanic White	190 (71.0)	220 (77.8)	207 (75.1)	268 (84.0)		28 (78.9)	33 (84.2)	35 (88.9)	31 (79.1)	
Other Hispanic	27 (4.7)	23 (3.0)	31 (4.1)	24 (2.8)		3 (1.9)	4 (4.4)	4 (4.8)	3 (4.9)	
Other race	24 (6.5)	23 (6.2)	26 (7.5)	16 (4.5)		8 (14.3)	2 (3.5)	1 (1.8)	3 (4.1)	
Education (*n* [%])					0.372					0.746
Below high school	138 (27.6)	129 (24.2)	156 (30.9)	142 (29.5)		14 (18.8)	11 (14.5)	12 (11.4)	12 (17.2)	
High school or above	224 (72.4)	252(75.8)	225 (69.1)	240 (70.5)		32 (81.2)	34 (85.5)	33 (88.6)	34 (82.8)	
Married (*n* [%])					0.026					0.748
No	143 (29.4)	155 (37.2)	169 (37.8)	185 (43.1)		15 (29.1)	10 (18.0)	12 (23.9)	14 (22.8)	
Yes	239 (70.6)	226 (62.8)	212 (62.2)	197 (56.9)		31 (70.9)	35 (82.0)	33 (76.1)	32 (77.2)	
Diabetes (*n* [%])					0.618					
No	245 (68.4)	234 (65.3)	248 (67.4)	251 (71.5)		46 (100.0)	45 (100.0)	45 (100.0)	46 (100.0)	
Yes	137 (31.6)	147 (34.7)	133 (32.6)	131 (28.5)						
Hypertension (*n* [%])					0.124					
No	337 (87.4)	324 (80.1)	316 (82.0)	332 (86.8)		46 (100.0)	45 (100.0)	45 (100.0)	46 (100.0)	
Yes	45 (12.6)	57 (19.9)	65 (18.0)	50 (13.2)						
Smoking (*n* [%])					0.44					
No	308 (75.6)	298 (77.4)	290 (74.2)	282 (70.4)		46 (100.0)	45 (100.0)	45 (100.0)	46 (100.0)	
Yes	74 (24.4)	83 (22.6)	91 (25.8)	100 (29.6)						
Drinking (*n* [%])					0.011					0.083
No	154 (36.8)	152 (31.2)	184 (45.1)	145 (36.3)		16 (20.8)	12 (21.4)	21 (46.9)	17 (31.6)	
Yes	228 (63.2)	229(68.8)	197 (54.9)	237 (63.7)		30 (79.2)	33 (78.6)	24 (53.1)	29 (68.4)	
CKD (*n* [%])					0.499					0.562
No	279 (76.9)	274 (71.4)	269 (75.3)	284 (72.2)		39 (82.8)	38 (79.3)	35 (73.3)	33 (66.0)	
Yes	103 (23.1)	107 (28.6)	112 (24.7)	98 (27.8)		7 (17.2)	7 (20.7)	10 (26.7)	13 (34.0)	
Hyperlipidemia (*n* [%])					0.259					
No	246 (67.7)	225 (60.6)	234 (59.4)	235 (58.9)		46 (100.0)	45 (100.0)	45 (100.0)	46 (100.0)	
Yes	136 (32.3)	156 (39.4)	147 (40.6)	247 (41.1)						
PIR	2.70 ± 1.60	2.53 ± 1.59	2.64 ± 1.58	2.48 ± 1.54	0.461	3.46 ± 1.57	3.31 ± 1.53	3.30 ± 1.51	2.89 ± 1.61	0.532
BMI (kg/m^2^)	29.93 ± 5.95	30.57 ± 6.10	30.15 ± 7.11	29.83 ± 7.62	0.625	26.59 ± 4.01	26.71 ± 3.28	28.01 ± 6.76	29.95 ± 6.30	0.102
LDL‐C (mmol/L)	2.57 ± 0.88	2.72 ± 1.04	2.72 ± 1.10	2.67 ± 1.03	0.243	2.27 ± 0.63	2.20 ± 0.59	2.23 ± 0.51	2.40 ± 0.57	0.565
HDL‐C (mmol/L)	1.30 ± 0.42	1.29 ± 0.42	1.29 ± 0.39	1.34 ± 0.48	0.608	1.40 ± 0.42	1.29 ± 0.29	1.38 ± 0.48	1.25 ± 0.32	0.482
TC (mmol/L)	4.57 ± 1.04	4.79 ± 1.20	4.74 ± 1.30	4.76 ± 1.17	0.088	4.23 ± 0.81	4.05 ± 0.69	4.25 ± 0.58	4.25 ± 0.66	0.531
TG (mmol/L)	1.52 ± 0.79	1.71 ± 0.90	1.59 ± 0.83	1.65 ± 0.83	0.251	1.22 ± 0.60	1.21 ± 0.63	1.39 ± 0.56	1.31 ± 0.67	0.606
ALT (U/L)	26.85±22.78	24.96±13.27	23.50 ± 15.49	22.86 ± 13.65	0.131	27.00 ± 11.85	25.82 ± 12.38	20.98 ± 6.51	21.31 ± 9.19	0.022
AST (U/L)	27.87 ± 21.58	25.41 ± 10.84	24.37 ± 9.85	24.45 ± 9.45	0.091	27.58 ± 8.15	26.16 ± 7.35	23.25 ± 4.71	23.31 ± 6.27	0.048
eGFR (mL/min/1.73m²)	101.60 ± 115.75	99.06 ± 103.53	101.65 ± 120.32	97.86 ± 97.94	0.96	77.83 ± 31.77	85.92 ± 34.92	87.51 ± 46.33	87.01 ± 44.71	0.608
SIRI	1.73 ± 0.92	2.07 ± 0.71	2.70 ± 1.12	3.85 ± 1.97	< 0.001	1.58 ± 0.59	2.14 ± 0.75	2.19 ± 0.85	3.49 ± 1.64	< 0.001
NLR	2.96 ± 0.49	3.55 ± 0.96	4.22 ± 1.34	6.06 ± 1.07	< 0.001	3.11 ± 0.57	3.47 ± 0.81	4.07± 1.55	5.51 ± 1.68	< 0.001
PLR	86.20 ± 102.97	116.47 ± 120.33	138.65 ± 115.17	204.21 ± 168.76	< 0.001	89.61 ± 98.11	112.99 ± 101.48	148.30 ± 121.00	206.19 ± 169.23	< 0.001

*Note*: ALT, alanine aminotransferase; AST, aspartate aminotransferase; SMuRF, standard modifiable risk factors.

Abbreviations: BMI, body mass index; CKD, chronic kidney disease; eGFR, estimated glomerular filtration rate; HDL‐C, high‐density lipoprotein cholesterol; LDL‐C, low‐density lipoprotein cholesterol; NLR, neutrophil‐to‐lymphocyte ratio; PIR, poverty‐to‐income ratio; PLR, platelet‐to‐lymphocyte ratio; SII, systemic inflammation index; SIRI, systemic inflammatory response index; TC, total cholesterol; TG, triglycerides.

**Table 2:** Baseline information according to quartiles of SIRI.

Variable	≥1 SMuRF				*p*	SMuRF‐less				*p*
	Q1 (*N* = 382)	Q2 (*N* = 381)	Q3 (*N* = 381)	Q4 (*N* = 382)		Q1 (*N* = 46)	Q2 (*N* = 45)	Q3 (*N* = 45)	Q4 (*N* = 46)	
SIRI	1.23 ± 0.27	1.88 ± 0.17	2.58 ± 0.25	4.50 ± 1.69	< 0.001	1.19 ± 0.29	1.81 ± 0.15	2.40 ± 0.24	3.96 ± 1.24	< 0.001
Age (years)	61.23 ± 13.04	64.74 ± 12.28	65.22 ± 12.07	68.68±11.30	< 0.001	58.11± 15.02	63.37 ± 13.22	66.66 ± 13.39	68.97 ± 11.75	0.051
Gender (*n* [%])					0.001					0.05
Male	183 (47.9)	223 (60.5)	241 (61.2)	266 (66.5)		26 (50.8)	36 (76.0)	37 (81.7)	37 (81.0)	
Female	135 (32.6)	158 (39.5)	140 (38.8)	116 (33.5)		20 (49.2)	9 (24.0)	8 (18.3)	9 (19.0)	
Race (*n* [%])					< 0.001					0.125
Mexican American	46 (5.4)	48 (4.0)	49 (3.5)	28 (3.3)		5 (4.7)	4 (2.4)	5 (5.7)	5 (5.9)	
Non‐Hispanic Black	116 (18.8)	66 (8.7)	50 (7.0)	34 (3.5)		5 (5.4)	1 (0.9)	2 (2.4)	0 (0.0)	
Non‐Hispanic White	161 (63.7)	207 (76.9)	237 (80.9)	280 (84.8)		25 (74.0)	35 (91.2)	32 (83.7)	35 (85.1)	
Other Hispanic	36 (5.5)	36 (4.3)	23 (3.0)	20 (2.0)		4 (3.8)	2 (1.1)	5 (7.6)	3 (3.0)	
Other race	23 (6.6)	24 (6.1)	22 (5.6)	20 (6.4)		7 (12.0)	3 (4.3)	1 (0.6)	3 (6.0)	
Education (*n* [%])					0.467					0.177
Below high school	136 (27.3)	147 (28.8)	145 (24.9)	137 (31.1)		12 (11.2)	14 (22.8)	11 (16.1)	12 (11.7)	
High school or above	246 (72.7)	234 (71.2)	236 (75.1)	245 (68.9)		34 (88.8)	30 (77.2)	34 (83.9)	34 (88.3)	
Married (*n* [%])					0.109					0.228
No	171 (40.0)	153 (32.3)	162 (41.4)	166 (34.9)		8 (15.3)	15 (30.0)	14 (30.6)	14 (18.8)	
Yes	211 (60.0)	228 (67.7)	219 (58.6)	216 (65.1)		38 (84.7)	30 (70.0)	31 (69.4)	32 (81.2)	
Diabetes (*n* [%])					0.398					
No	251 (70.2)	239 (68.4)	242 (63.8)	246 (70.5)		46 (100.0)	45 (100.0)	45 (100.0)	46 (100.0)	
Yes	131 (29.8)	142 (31.6)	139 (36.2)	136 (29.5)						
Hypertension (*n* [%])					0.382					
No	320 (83.4)	322 (80.7)	329 (84.9)	338 (86.9)		46 (100.0)	45 (100.0)	45 (100.0)	46 (100.0)	
Yes	62 (16.6)	59 (19.3)	52 (15.1)	44 (13.1)						
Smoking (*n* [%])					0.386					
No	299 (75.6)	298 (74.9)	299 (77.0)	282 (70.2)		46 (100.0)	45 (100.0)	45 (100.0)	46 (100.0)	
Yes	83 (24.4)	83 (25.1)	82 (23.0)	100 (29.8)						
Drinking (*n* [%])					0.802					0.586
No	170 (37.9)	158 (38.5)	149 (34.7)	158 (38.5)		18 (34.5)	13 (23.8)	15 (25.6)	20 (38.9)	
Yes	212 (62.1)	223 (61.5)	232 (65.3)	224 (61.5)		28 (65.5)	32 (76.2)	30 (74.4)	26 (61.1)	
CKD (*n* [%])					0.117					0.755
No	255 (67.6)	273 (74.5)	281 (78.1)	297 (74.4)		36 (71.6)	36 (76.5)	38 (83.0)	35 (71.5)	
Yes	127 (32.4)	108 (25.5)	100 (21.9)	85 (25.6)		10 (28.4)	9 (23.5)	7 (17.0)	11 (28.5)	
Hyperlipidemia (*n* [%])					0.166					
No	239 (65.6)	232 (61.6)	222 (55.4)	247 (64.0)		46 (100.0)	45 (100.0)	45 (100.0)	46 (100.0)	
Yes	143 (34.4)	149 (38.4)	159 (44.6)	135 (36.0)						
PIR	2.57 ± 1.68	2.54 ± 1.53	2.61 ± 1.67	2.61 ± 1.44	0.959	3.35 ± 1.59	3.28 ± 1.45	3.15 ± 1.61	3.26 ± 1.59	0.966
BMI (kg/m^2^)	29.99±6.57	30.57±6.10	30.53±7.22	29.37±6.89	0.153	26.07±3.93	29.20 ± 6.37	27.21 ± 4.81	28.80 ± 6.05	0.107
LDL‐C (mmol/L)	2.93 ± 1.07	2.71 ± 1.00	2.63 ± 1.00	2.45 ± 0.97	< 0.001	2.29 ± 0.55	2.25 ± 0.58	2.30 ± 0.56	2.24 ± 0.64	0.976
HDL‐C (mmol/L)	1.33±0.39	1.32±0.43	1.27±0.43	1.31±0.46	0.527	1.57±0.51	1.24±0.29	1.25 ± 0.29	1.25±0.32	0.059
TC (mmol/L)	4.98 ± 1.21	4.79 ± 1.20	4.68 ± 1.15	4.47± 1.12	< 0.001	4.34 ± 0.70	4.19 ± 0.78	4.12 ± 0.56	4.11±0.70	0.503
TG (mmol/L)	1.58 ± 0.87	1.65 ± 0.82	1.71 ± 0.87	1.53 ± 0.80	0.114	1.05 ± 0.52	1.53 ± 0.70	1.24 ± 0.50	1.37± 0.64	0.606
ALT (U/L)	24.79 ± 14.94	24.54 ± 12.71	23.50 ± 15.49	22.56 ± 19.53	0.172	24.67 ± 13.62	25.40 ± 8.75	22.08 ± 8.40	23.01 ± 9.80	0.036
AST (U/L)	25.16 ± 12.08	25.61 ± 10.76	26.12 ± 12.01	24.87 ± 18.35	0.759	25.27 ± 8.67	27.16 ± 6.13	23.61 ± 5.08	24.44 ± 6.79	0.433
eGFR (mL/min/1.73 m²)	87.12 ± 96.59	95.00 ± 87.46	105.36 ± 129.63	110.42 ± 116.47	0.008	79.02 ± 36.29	77.62 ± 35.76	91.76 ± 42.00	90.07 ± 44.16	0.083
SII	686.83 ± 249.67	778.03 ± 263.98	972.03 ± 316.68	1428.62 ± 832.58	< 0.001	643.2 ± 248.08	766.97 ± 219.92	822.40 ± 282.73	1222.45 ± 554.85	< 0.001
NLR	2.94 ± 0.91	3.57 ± 1.45	4.14 ± 3.04	6.13 ± 4.55	< 0.001	3.06 ± 0.48	3.56 ± 0.97	3.92 ± 1.22	5.63 ± 6.09	< 0.001
PLR	119.39 ± 105.33	123.46 ± 120.17	133.47 ± 119.96	169.21 ± 132.47	< 0.001	127.23 ± 82.05	127.91 ± 101.56	124.06 ± 96.00	177.70 ± 134.21	< 0.001

*Note*: ALT, alanine aminotransferase; AST, aspartate aminotransferase; SMuRF, standard modifiable risk factors.

Abbreviations: BMI, body mass index; CKD, chronic kidney disease; eGFR, estimated glomerular filtration rate; HDL‐C, high‐density lipoprotein cholesterol; LDL‐C, low‐density lipoprotein cholesterol; NLR, neutrophil‐to‐lymphocyte ratio; PIR, poverty‐to‐income ratio; PLR, platelet‐to‐lymphocyte ratio; SII, systemic inflammation index; SIRI, systemic inflammatory response index; TC, total cholesterol; TG, triglycerides.

### 3.2. Associations Between SII/SIRI and Mortality in Patients With ≥1 SMuRF and SMuRF‐Less Groups

Schoenfeld’s residual test indicated global *p*‐values of 0.128 and 0.234 for all‐cause mortality and cardiovascular mortality endpoints, respectively, suggesting that the proportional hazards assumption was met. Multivariate Cox regression showed that after adjusting for potential covariates, in patients with ≥1 SMuRF, the SII Q4 and SIRI Q4 group were strongly associated with an increase in both all‐cause mortality (SII: hazard ratio [HR] 1.47, 95% confidence interval [CI] 1.18–1.84, *p*  < 0.001; SIRI:HR 1.66, 95%CI 1.31–2.10, *p*  < 0.001, Table 3) and cardiovascular mortality (SII:HR 1.52, 95%CI 1.07–2.17, *p* = 0.020; SIRI:HR 1.63, 95%CI 1.11–2.38, *p* = 0.011, Table S1) compared with the SII Q1 and SIRI Q1 group, respectively. *p*‐Value trend suggested a linear association between SII, SIRI and both all‐cause and cardiovascular mortality (all *p* trend < 0.05, Table 3, Table S1). Additionally, we observed that in patients with ≥1 SMuRF, NLR and PLR were linearly associated with all‐cause mortality and cardiovascular mortality (all trend *p*‐values < 0.05, Table S2, Table S3).

**Table 3:** Association between SII/SIRI and all‐cause mortality in patients with coronary atherosclerosis, stratified by SMuRF status.

Character	HR (95% CI)	*p*	*p* trend	HR (95% CI)	*p*	*p* trend	HR (95%CI)	*p*	*p* trend
≥ 1SMuRF									
SII (tertile)			< 0.001			< 0.001			< 0.001
Q1	ref.	ref.		ref.	ref.		ref.	ref.	
Q2	1.04 (0.82,1.31)	0.713		1.01(0.79,1.26)	0.956		1.07 (0.84,1.34)	0.586	
Q3	1.12 (0.89,1.40)	0.320		1.17(0.93,1.47)	0.160		1.21 (0.96,1.52)	0.094	
Q4	1.45 (1.17,1.80)	< 0.001		1.39(1.12,1.74)	0.002		1.47 (1.18,1.84)	< 0.001	
SIRI (tertile)			< 0.001			< 0.001			< 0.001
Q1	ref.	ref.		ref.	ref.		ref.	ref.	
Q2	1.35 (1.07,1.71)	0.011		1.14 (0.90,1.45)	0.267		1.12 (0.88,1.43)	0.338	
Q3	1.54 (1.22,1.94)	< 0.001		1.14 (0.91,1.44)	0.252		1.11 (0.87,1.40)	0.396	
Q4	2.61 (2.09,3.25)	< 0.001		1.75 (1.39,2.22)	< 0.001		1.66 (1.31,2.10)	< 0.001	
SMuRF‐less									
SII (tertile)			< 0.001			< 0.001			< 0.001
Q1	ref.	ref.		ref.	ref.		ref.	ref.	
Q2	1.34 (0.61,2.91)	0.461		1.51 (0.66,3.43)	0.327		1.50 (0.65,3.49)	0.343	
Q3	1.15 (0.54,2.47)	0.714		1.74 (0.77,3.96)	0.185		1.70 (0.73,3.93)	0.219	
Q4	2.05 (1.02,4.10)	0.043		3.33 (1.52,7.26)	0.002		3.32 (1.45,7.59)	0.004	
SIRI (tertile)			< 0.001			< 0.001			0.016
Q1	ref.	ref.		ref.	ref.		ref.	ref.	
Q2	1.70 (0.70,4.09)	0.239		1.33 (0.52,3.38)	0.550		1.62 (0.61,4.28)	0.326	
Q3	2.03 (0.88,4.71)	0.098		1.64 (0.67,4.03)	0.281		2.13 (0.81,5.60)	0.123	
Q4	4.39 (1.99,9.68)	< 0.001		4.16 (1.76,9.81)	0.001		4.25 (1.67,10.80)	0.002	

*Note:* Crude model: univariable model. Model 1: covariates were adjusted for age, gender, race, education level, marital status, poverty‐to‐income ratio (PIR), drinking, smoking, body mass index (BMI), hypertension, diabetes, and chronic kidney disease (CKD). Model 2: covariates were adjusted for the same variables as Model 2 as well as low‐density lipoprotein cholesterol (LDL‐C), high‐density lipoprotein cholesterol (HDL‐C), total cholesterol (TC), triglycerides (TG), alanine aminotransferase (ALT), and aspartate aminotransferase (AST). SMuRF, standard modifiable risk factors.

Abbreviations: SII, systemic inflammation index; SIRI, systemic inflammatory response index.

In patients with SMuRF‐less, the risk of all‐cause mortality was also significantly higher in the SII Q4 and SIRI Q4 group compared with the SII Q1 and SIRI Q1 group, respectively (SII:HR 3.32, 95%CI 1.45–7.59, *p* = 0.004; SIRI:HR 4.25, 95%CI 1.67–10.80, *p* = 0.002, Table 3). Besides, there was also a linear positive correlation between SII, SIRI and all‐cause mortality (SII:*p* trend < 0.001; SIRI:*p* trend = 0.016, Table 3). In terms of cardiovascular mortality, there was a positive correlation between SIRI and cardiovascular mortality (SIRI:HR 11.69, 95%CI 1.43–95.21, *p* = 0.028, Table S1), whereas there was no significant association with SII (SII:HR 2.21, 95%CI 0.54–8.97, *p* = 0.272, Table S1). *p*‐Value trend revealed that neither SII, nor SIRI is linearly related to cardiovascular mortality (SII:*p* trend = 0.060, SIRI:*p* trend = 0.060, Table S1). After adjusting for potential covariates, no significant association was observed between NLR, PLR, and all‐cause mortality or cardiovascular mortality (Table S2, Table S3).

Similarly, Kaplan–Meier survival curves revealed that in terms of all‐cause mortality, in both groups of patients, the group with lower levels of SII and SIRI had a higher long‐term survival rate than the group with higher levels (*p*  < 0.001, Figure 2), although the difference in survival in patients with SMuRF‐less was not statistically significant for SII (*p* = 0.14, Figure 2). As for cardiovascular mortality, among patients with ≥1 SMuRFs, patients with lower levels of SII and SIRI still had higher long‐term survival than those in the other groups (SII: *p* = 0.002, SIRI:*p*  < 0.001, Figure S1), but there was no statistically significant difference in survival between the SII and SIRI groups in patients with SMuRF‐less (SII:*p* = 0.12, SIRI:*p* = 0.052, Figure S1).

Figure 2Kaplan–Meier curves for all‐cause death during follow‐up for patients in each subgroup. (A) Kaplan–Meier curves of patients with SMuRFs grouped according to SII; (B) Kaplan–Meier curves of patients with SMuRFs grouped according to SIRI; (C) Kaplan–Meier curves of patients with SMuRF‐less grouped according to SII; (D) Kaplan–Meier curves of patients with SMuRF‐less grouped according to SIRI.

**Figure:**
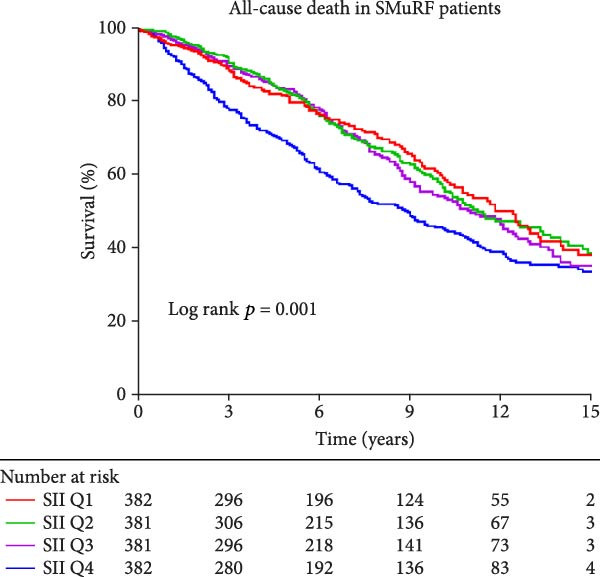
(A)

**Figure:**
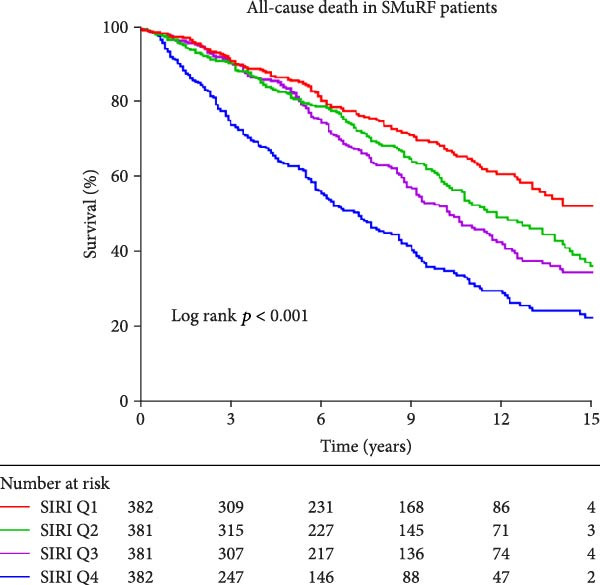
(B)

**Figure:**
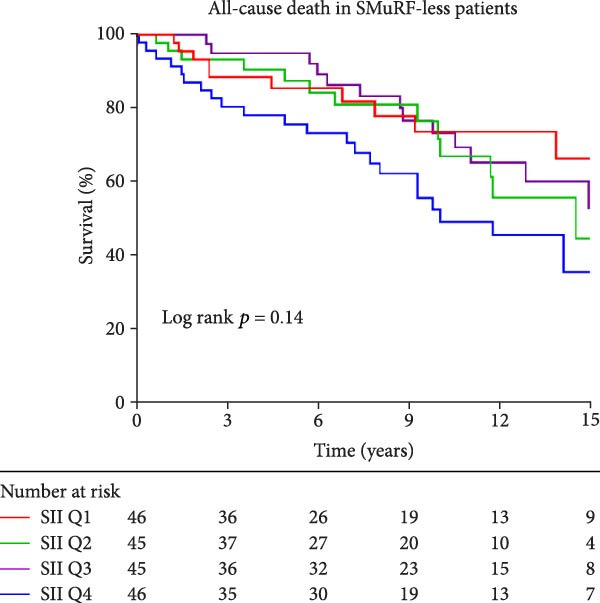
(C)

**Figure:**
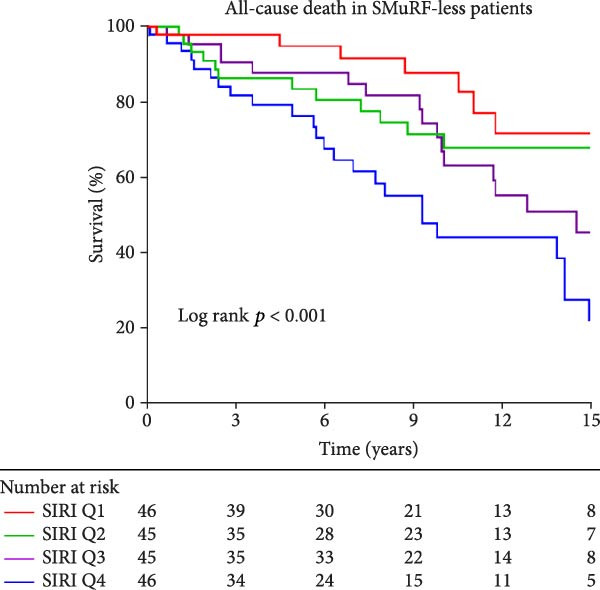
(D)

RCS analysis was performed to assess the potential dose–response relationship between SII, SIRI and mortality. In Figure 3, a positive correlation between SII, SIRI and all‐cause mortality was found in both groups (*p*‐overall < 0.05). Of note, SIRI showed a nonlinear relationship with all‐cause mortality in patients with SMuRF‐less (*p*‐nonlinear = 0.013), while the rest showed a linear relationship (*p*‐nonlinear > 0.05). As for cardiovascular mortality, SII and SIRI still showed linear trend in patients with ≥1 SMuRF (*p*‐overall < 0.05, *p*‐nonlinear > 0.05), but no significant trend in patients with SMuRF‐less (*p*‐overall > 0.05) (Figure S2).

Figure 3Relationship between SII/SIRI and all‐cause mortality as assessed by the RCS after correction for covariates. (A) RCS analysis of patients with SMuRFs grouped according to SII; (B) RCS analysis of patients with SMuRFs grouped according to SIRI; (C) RCS analysis of patients with SMuRF‐less grouped according to SII; (D) RCS analysis of patients with SMuRF‐less grouped according to SIRI. The solid blue line corresponds to the central estimates and the light blue shading indicates the 95% confidence intervals. RCS, restricted cubic spline; SII, systemic inflammation index; SIRI, systemic inflammatory response index; SMuRF, standard modifiable risk factors.

**Figure:**
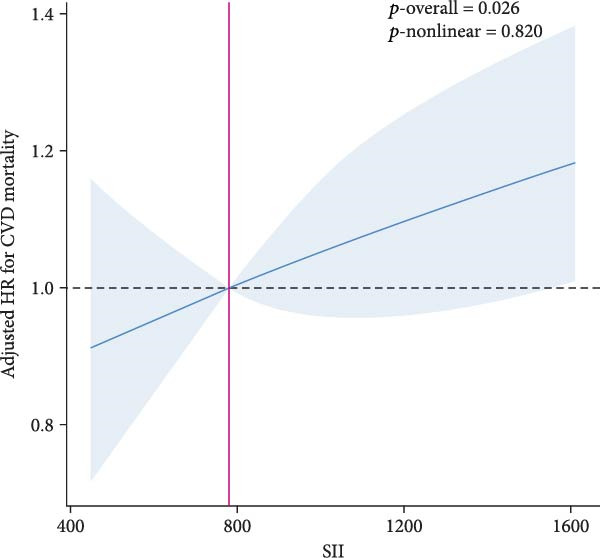
(A)

**Figure:**
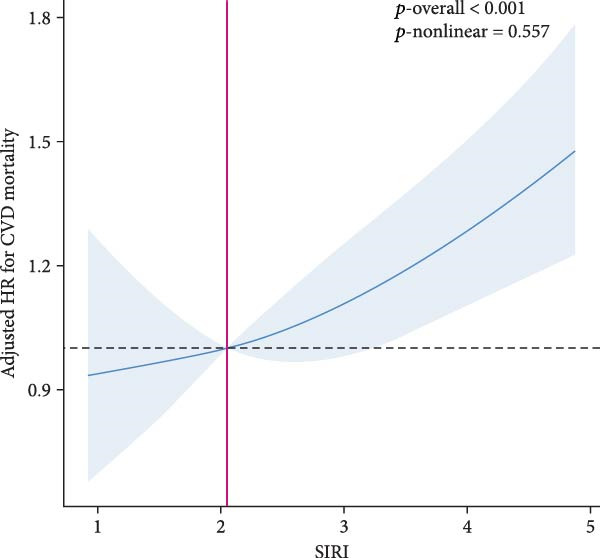
(B)

**Figure:**
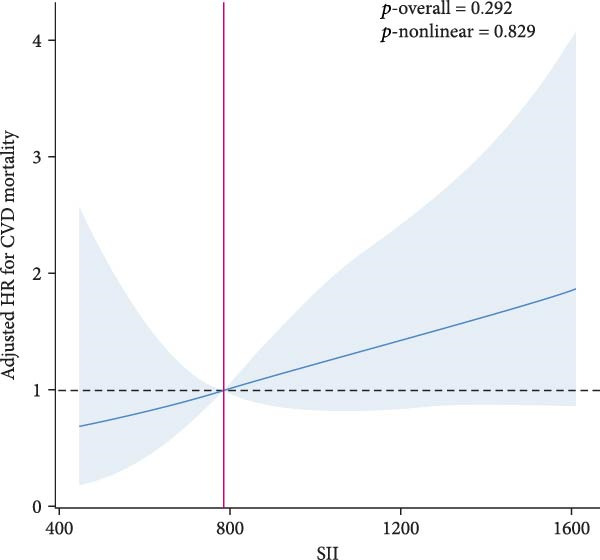
(C)

**Figure:**
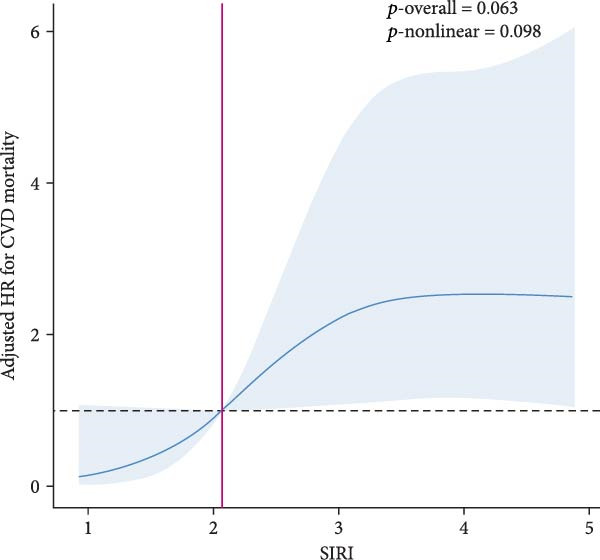
(D)

## 4. Discussion

Inflammation is recognized as a potential factor in the development of many diseases, including CHD. Inflammation in the human body promotes an abnormal elevation of platelets, while the abnormally aggregated platelets adhere to the surface of vascular endothelial cells, inducing local ischemia, hypoxia, and microthrombosis, which results in vascular occlusion, causing malignant events such as MI, stroke, and peripheral vascular disease [19]. Previous studies have shown that a variety of inflammatory markers in the body, such as CRP and interleukin‐6, have been shown to have prognostic significance in patients with coronary artery disease [20–22]. Recently, SII and SIRI have been proposed as emerging inflammatory markers that are able to respond to the degree of inflammatory response in a much simpler and quicker way. SII and SIRI have been demonstrated to have a strong correlation with the occurrence of ACS in patients with coronary artery disease [12, 23].

However, it should be noted that chronic inflammation is often a common pathway for multiple cardiovascular risk factors to mediate coronary atherosclerosis. Specifically, high levels of lipoprotein(a) promote arterial wall inflammation and increased migration of monocytes into atherosclerotic plaques, with long‐term effects on coronary inflammatory plaque formation [24], in addition to insulin resistance, which can mediate coronary artery disease through inflammatory responses [25]. Previous studies have tended to focus more on the prognostic impact of inflammation in populations of patients with coronary artery disease in the presence of traditional risk factors [26], however, the role of inflammation in patients without specific risk factors is not clear.

First and foremost, it is important to emphasize that, given the observational design of the NHANES survey, our findings demonstrate a strong association but cannot establish a causal relationship between elevated SII/SIRI and mortality. Our results should be interpreted as hypothesis‐generating in this context. In our study, we divided patients with CHD into two groups according to the presence of traditional risk factors, and after adjusting for relevant confounders, we found that both SII and SIRI were linearly and significantly associated with all‐cause mortality and cardiovascular mortality in the SMuRF ≥1 population, which is consistent with the findings of a previous study [27]. In contrast, in patients with SMuRF‐less, we found significant associations between both inflammatory indices and all‐cause mortality, and RCS analysis suggested that SII was linearly associated with all‐cause mortality, whereas SIRI was nonlinearly associated. Notably, compared to established inflammatory markers NLR and PLR, SII and SIRI demonstrated stronger associations with all‐cause mortality and cardiovascular mortality in patients with SMuRF‐less. This suggests potential clinical value for SII and SIRI in this population, warranting further validation through prospective studies. To our knowledge, this is the first study to specifically examine the association of SII and SIRI with long‐term clinical outcomes in patients with SMuRF ≥ 1 and SMuRF‐less. Our results suggest that high levels of SII and SIRI are strongly associated with all‐cause mortality regardless of the presence of traditional risk factors. This suggests that we need to pay more attention to the role of inflammation in atherosclerosis, controlling the development of inflammation is particularly important in patients who are not diabetic, hypertensive, or nonsmokers and has the potential to improve their prognosis and survival.

The question of why SMuRF‐less patients exhibit elevated systemic inflammation is a critical area for future research. Several plausible mechanisms, not captured by standard risk factor assessment, could be involved. A genetic predisposition to a heightened inflammatory response or dysregulated immune function may play a significant role. Furthermore, a range of nontraditional or residual risk factors could contribute, including chronic psychosocial stress, exposure to environmental pollutants like air pollution, pro‐inflammatory dietary patterns, gut dysbiosis, or subclinical chronic infections such as periodontal disease. These factors could collectively promote a state of chronic low‐grade inflammation, thereby driving atherogenesis and increasing mortality risk independently of conventional risk factors.

In patients with cirrhosis and stroke, the hyperinflammatory response is also an important factor in disease progression [28, 29]. Similarly, in CHD patients, the severity of MI can be reduced, and cardiac function can be improved by decreasing the myocardial inflammatory response [30]. Our study demonstrated that patients with a high inflammatory response had lower long‐term survival than patients with lower levels of inflammation, irrespective of whether they had risk factors or not. Therefore, in CHD patients, control of the inflammatory response becomes more important than just control of blood pressure, blood glucose, and lipids. Several recent studies have exacerbated the inflammatory response by modulating macrophage polarization in a mouse model of MI, all of which ultimately affected cardiac function and survival after MI in mice [31, 32]. In contrast, attenuating the inflammatory response improves cardiac function and remodeling after MI [33]. In recent years, many clinical studies have found that several treatments targeting the risk of residual inflammation can significantly reduce the rate of vascular events [34, 35]. There is evidence that low‐dose colchicine in combination with statins safely reduces major adverse cardiovascular events in patients with stable atherosclerosis, with a greater magnitude of benefit than continuous lipid‐lowering agents alone [36]. However, most of the current studies have been conducted only in patients with ≥1 SMuRF, and it is unclear whether the residual risk of inflammation can be reduced by anti‐inflammatory agents to improve patient prognosis in patients with SMuRF‐less. Our study reveals that inflammation is an important factor influencing the prognosis of patients with CHD and is independent of traditional cardiovascular risk factors; therefore, anti‐inflammatory therapy may remain a potential therapeutic target for such patients.

Our study has several limitations. First, diagnosis of cardiovascular disease is confirmed by self‐report, which may have some false positives or missed patients with unreported cardiovascular disease. Second, the number of patients in the SMuRF‐less subgroup (*n* = 182) was relatively small, and an a priori power calculation was not performed as this was a post hoc analysis of a public database. This limited statistical power may have reduced our ability to detect significant associations, particularly for outcomes with fewer events like cardiovascular mortality. For instance, the lack of a significant association between SII and cardiovascular mortality in this group (*p* = 0.272) should be interpreted with caution, as it may represent a Type II error. Therefore, our findings in the SMuRF‐less population should be considered exploratory, and validation in larger, dedicated cohorts is essential. Third, more research is needed to determine if these findings can be widely used in other areas.

## 5. Conclusions

Taken together, findings in this study showed that in CHD patients with ≥1 SMuRF, SII and SIRI were positively correlated with all‐cause mortality and cardiovascular mortality. While in CHD patients with SMuRF‐less, higher levels of SII and SIRI also significantly increase the prevalence of all‐cause mortality, but not with cardiovascular mortality. This suggests that inflammation may be an important factor contributing to poor prognosis independently of atherosclerosis‐specific risk factors, and further studies are needed to better explore their association and underlying causal mechanisms.

**Nomenclature**
SMuRFs: Standard modifiable risk factors
SII: Systemic immune inflammatory index
SIRI: Systemic inflammatory response index
NHANES: National Health and Nutrition Examination Survey
CHD: Coronary heart disease
RCS: Restricted cubic spline
ACS: Acute coronary syndrome
STEMI: ST‐segment elevation myocardial infarction
NLR: Neutrophil to lymphocyte ratio
PLR: Platelet‐to‐lymphocyte ratio
CDC: Centers for Disease Control and Prevention
NCHS: National Center for Health Statistics
Ldl‐c: Low‐density lipoprotein cholesterol
FBG: Fasting blood glucose
SBP: Systolic blood pressure
DBP: Diastolic blood pressure
PIR: Poverty‐to‐income ratio
BMI: Body mass index
TC: Total cholesterol
TG: Triglycerides
HDL‐C: High‐density lipoprotein cholesterol
ALT: Alanine aminotransferase
AST: Aspartate aminotransferase
eGFR: Estimated glomerular filtration rate
CKD: Chronic kidney disease
PSUs: Primary sampling units
CVDs: Cardiovascular diseases
MI: Myocardial infarction.

## Ethics Statement

The data are publicly available (in the NHANES database); hence, ethical approval statement and informed consent are not required for the study.

## Conflicts of Interest

The authors declare no conflicts of interest.

## Author Contributions

Weiren Yan, Bingqian Zhang, and Xiaoyan Zhang contributed equally to this work.

## Funding

This work is supported by the National Natural Science Foundation of China (Grant 82170252), the Liaoning Revitalization Talents Program (Grant XLYC2203054), the Innovation Team Project of Higher Education Institutions in Liaoning Province (Grant LJ222410161084), the Outstanding Youth Scientific Talent Project of Dalian (Grant 2024RJ013), and the Dalian Science and Technology Innovation Fundation (Grant 2023JJ13SN039).

## Supporting Information

Additional supporting information can be found online in the Supporting Information section.

## Supporting information


**Supporting Information** Table S1. Association between SII/SIRI and Cardiovascular Mortality in Patients with Coronary Atherosclerosis, Stratified by SMuRF Status. Table S2. Association between NLR/PLR and All‐cause Mortality in Patients with Coronary Atherosclerosis, Stratified by SMuRF Status. Table S3. Association between NLR/PLR and Cardiovascular Mortality in Patients with Coronary Atherosclerosis, Stratified by SMuRF Status. Figure S1. Kaplan‐Meier curves for cardiovascular death during follow‐up for patients in each subgroup. Figure S2. Relationship between SII/SIRI and cardiovascular mortality as assessed by the RCS after correction for covariates.

## Data Availability

The laboratory data from our study are publicly accessible online at https://wwwn.cdc.gov/nchs/nhanes/Default.aspx for global data users and researchers.
