# Supplementary material for: Association of Systemic Inflammation Indices With Mortality in Coronary Atherosclerosis Patients With and Without Standard Modifiable Risk Factors
Source: Mediators Inflamm. 2026 Jan 10;2026:8830121. doi: 10.1155/mi/8830121 (PMC12790178; doi:10.1155/mi/8830121)
Supplement: Supplementary file 1 — Supporting Information Table S1. Association between SII/SIRI and Cardiovascular Mortality in Patients with Coronary Atherosclerosis, Stratified by SMuRF Status. Table S2. Association between NLR/PLR and All‐cause Mortality in Patients with Coronary Atherosclerosis, Stratified by SMuRF Status. Table S3. Association between NLR/PLR and Cardiovascular Mortality in Patients with Coronary Atherosclerosis, Stratified by SMuRF Status. Figure S1. Kaplan‐Meier curves for cardiovascular death during follow‐up for patients in each subgroup. Figure S2. Relationship between SII/SIRI and cardiovascular mortality as assessed by the RCS after correction for covariates. [file MI-2026-8830121-s001.docx]

**Supplementary table 1. Association between SII/SIRI and Cardiovascular Mortality in Patients with Coronary Atherosclerosis, Stratified by SMuRF Status.**

|  | Crude model | | | Model1 | | | Model2 | | |
| --- | --- | --- | --- | --- | --- | --- | --- | --- | --- |
| Character | HR (95%CI) | P | P trend | HR (95%CI) | P | P trend | HR (95%CI) | P | P trend |
| **≥1SMuRF** | | | | | | | | | |
| **SII (Tertile)** |  |  | **0.001** |  |  | **0.011** |  |  | **0.010** |
| Q1 | ref. | ref. |  | ref. | ref. |  | ref. | ref. |  |
| Q2 | 0.85 (0.57,1.24) | 0.304 |  | 1.01 (0.56,1.21) | 0.320 |  | 0.89 (0.61,1.32) | 0.570 |  |
| Q3 | 0.89 (0.61,1.30) | 0.532 |  | 1.17 (0.64,1.38) | 0.758 |  | 0.99 (0.67,1.46) | 0.961 |  |
| Q4 | 1.49 (1.06,2.09) | **0.023** |  | 1.39 (0.98,2.01) | 0.050 |  | 1.52 (1.07,2.17) | **0.020** |  |
| **SIRI (Tertile)** |  |  | **<0.001** |  |  | **<0.001** |  |  | **<0.001** |
| Q1 | ref. | ref. |  | ref. | ref. |  | ref. | ref. |  |
| Q2 | 1.24 (0.84,1.84) | 0.293 |  | 1.06 (0.71,1.58) | 0.787 |  | 1.05 (0.69,1.56) | 0.832 |  |
| Q3 | 1.36 (0.92,2.01) | 0.120 |  | 1.02 (0.69,1.52) | 0.914 |  | 0.96 (0.69,1.56) | 0.837 |  |
| Q4 | 2.74 (1.91,3.92) | **<0.001** |  | 1.77 (1.21,2.58) | **0.003** |  | 1.63 (1.11,2.38) | **0.011** |  |
| **SMuRF-less** | | | | | | | | | |
| **SII (Tertile)** |  |  | **0.006** |  |  | **0.040** |  |  | 0.060 |
| Q1 | ref. | ref. |  | ref. | ref. |  | ref. | ref. |  |
| Q2 | 0.40 (0.08, 2.02) | 0.273 |  | 0.50 (0.09,2.67) | 0.427 |  | 0.58 (0.10,3.29) | 0.549 |  |
| Q3 | 0.31 (0.06,1.58) | 0.362 |  | 0.44 (0.08,2.43) | 0.350 |  | 0.66 (0.10,4.03) | 0.652 |  |
| Q4 | 1.38 (0.47,4.01) | 0.556 |  | 1.90 (0.53,6.70) | 0.322 |  | 2.21 (0.54,8.97) | 0.272 |  |
| **SIRI (Tertile)** |  |  | **<0.001** |  |  | **0.020** |  |  | 0.060 |
| Q1 | ref. | ref. |  | ref. | ref. |  | ref. | ref. |  |
| Q2 | 1.57 (0.26,9.42) | 0.621 |  | 1.33 (0.20,8.82) | 0.761 |  | 2.17 (0.26,17.84) | 0.470 |  |
| Q3 | 2.32 (0.44,11.99) | 0.326 |  | 2.11 (0.36,12.32) | 0.412 |  | 5.15 (0.61,43.38) | 0.134 |  |
| Q4 | 5.47 (1.15,25.98) | **0.034** |  | 5.40 (1.02,28.56) | **0.043** |  | 11.69 (1.43,95.21) | **0.028** |  |

Crude model: univariable model

Model 1: covariates were adjusted for age, gender, race, education level, marital status, poverty-to-income ratio (PIR), drinking, smoking, body mass index (BMI), hypertension, diabetes, chronic kidney disease (CKD)

Model 2: covariates were adjusted for the same variables as model2 as well as low-density lipoprotein cholesterol (LDL-C), high-density lipoprotein Abbreviations: cholesterol (HDL-C), total cholesterol (TC), triglycerides (TG), alanine aminotransferase (ALT), aspartate aminotransferase (AST)

SMuRF, standard modifiable risk factors; SII, systemic inflammation index; SIRI, systemic inflammatory response index

**Supplementary table 2. Association between NLR/PLR and All-cause Mortality in Patients with Coronary Atherosclerosis, Stratified by SMuRF Status.**

|  | Crude model | | | Model1 | | | Model2 | | |
| --- | --- | --- | --- | --- | --- | --- | --- | --- | --- |
| Character | HR (95%CI) | P | P trend | HR (95%CI) | P | P trend | HR (95%CI) | P | P trend |
| **≥1SMuRF** | | | | | | | | | |
| **NLR (Tertile)** |  |  | **0.001** |  |  | **<0.001** |  |  | **0.020** |
| Q1 | ref. | ref. |  | ref. | ref. |  | ref. | ref. |  |
| Q2 | 1.12 (0.89,1.40) | 0.322 |  | 1.14 (0.91,1.44) | 0.240 |  | 1.15 (0.91,1.45) | 0.225 |  |
| Q3 | 1.18 (0.94,1.48) | 0.138 |  | 1.22 (0.97,1.53) | 0.081 |  | 1.22 (0.97,1.54) | 0.076 |  |
| Q4 | 1.49 (1.06,2.09) | **<0.001** |  | 1.75 (1.40,2.18) | **<0.001** |  | 1.76 (1.41,2.20) | **<0.001** |  |
| **PLR (Tertile)** |  |  | **<0.001** |  |  | **0.002** |  |  | **<0.001** |
| Q1 | ref. | ref. |  | ref. | ref. |  | ref. | ref. |  |
| Q2 | 1.36 (1.07,1.72) | **0.010** |  | 1.37 (1.08,1.74) | **0.008** |  | 1.15 (0.91,1.45) | 0.225 |  |
| Q3 | 1.47 (1.16,1.86) | **0.001** |  | 1.47 (1.17,1.87) | **0.001** |  | 1.22 (0.97,1.54) | 0.076 |  |
| Q4 | 2.39 (1.92,2.98) | **<0.001** |  | 2.38 (1.90,2.99) | **<0.001** |  | 1.76 (1.41,2.20) | **<0.001** |  |
| **SMuRF-less** | | | | | | | | | |
| **NLR(Tertile)** |  |  | **0.040** |  |  | 0.375 |  |  | 0.450 |
| Q1 | ref. | ref. |  | ref. | ref. |  | ref. | ref. |  |
| Q2 | 0.48 (0.25, 0.89) | 0.020 |  | 0.55 (0.28,1.07) | 0.079 |  | 0.51 (0.26,1.02) | 0.057 |  |
| Q3 | 0.66 (0.34,1.27) | 0.218 |  | 0.82 (0.40,1.68) | 0.597 |  | 0.86 (0.41,1.81) | 0.709 |  |
| Q4 | 1.12 (1.04,1.29) | **0.034** |  | 1.45 (0.30,1.63) | 0.408 |  | 1.22 (0.66,1.92) | 0.640 |  |
| **PLR (Tertile)** |  |  | **0.004** |  |  | **0.020** |  |  | 0.060 |
| Q1 | ref. | ref. |  | ref. | ref. |  | ref. | ref. |  |
| Q2 | 1.12 (0.62,2.04) | 0.687 |  | 1.13 (0.61,2.06) | 0.690 |  | 0.94 (0.50,1.76) | 0.853 |  |
| Q3 | 0.85 (0.40,1.82) | 0.687 |  | 0.79 (0.37,1.71) | 0.564 |  | 0.85 (0.39,1.84) | 0.684 |  |
| Q4 | 1.43 (1.03,2.01) | **0.002** |  | 1.33 (1.20,2.02) | **0.021** |  | 1.32 (0.78,2.06) | 0.089 |  |

Crude model: univariable model

Model 1: covariates were adjusted for age, gender, race, education level, marital status, poverty-to-income ratio (PIR), drinking, smoking, body mass index (BMI), hypertension, diabetes, chronic kidney disease (CKD)

Model 2: covariates were adjusted for the same variables as model2 as well as low-density lipoprotein cholesterol (LDL-C), high-density lipoprotein Abbreviations: cholesterol (HDL-C), total cholesterol (TC), triglycerides (TG), alanine aminotransferase (ALT), aspartate aminotransferase (AST)

SMuRF, standard modifiable risk factors; NLR, neutrophil-to-lymphocyte ratio; PLR, platelet-lymphocyte ratio

**Supplementary table 3. Association between NLR/PLR and Cardiovascular Mortality in Patients with Coronary Atherosclerosis, Stratified by SMuRF Status.**

Crude model: univariable model

Model 1: covariates were adjusted for age, gender, race, education level, marital status, poverty-to-income ratio (PIR), drinking, smoking, body mass index (BMI), hypertension, diabetes, chronic kidney disease (CKD)

Model 2: covariates were adjusted for the same variables as model2 as well as low-density lipoprotein cholesterol (LDL-C), high-density lipoprotein Abbreviations: cholesterol (HDL-C), total cholesterol (TC), triglycerides (TG), alanine aminotransferase (ALT), aspartate aminotransferase (AST)

SMuRF, standard modifiable risk factors; NLR, neutrophil-to-lymphocyte ratio; PLR, platelet-lymphocyte ratio

|  | Crude model | | | Model1 | | | Model2 | | |
| --- | --- | --- | --- | --- | --- | --- | --- | --- | --- |
| Character | HR (95%CI) | P | P trend | HR (95%CI) | P | P trend | HR (95%CI) | P | P trend |
| **≥1SMuRF** | | | | | | | | | |
| **NLR (Tertile)** |  |  | **<0.001** |  |  | **<0.001** |  |  | **<0.001** |
| Q1 | ref. | ref. |  | ref. | ref. |  | ref. | ref. |  |
| Q2 | 1.22 (0.83,1.80) | 0.306 |  | 1.23 (0.83,1.82) | 0.293 |  | 1.23 (0.83,1.82) | 0.290 |  |
| Q3 | 1.29 (0.88,1.88) | 0.188 |  | 1.33 (0.90,1.96) | 0.139 |  | 1.35 (0.91,1.98) | 0.127 |  |
| Q4 | 1.91 (1.33,2.73) | **<0.001** |  | 1.90 (1.31,2.76) | **<0.001** |  | 1.93 (1.32,2.80) | **<0.001** |  |
| **PLR (Tertile)** |  |  | **<0.001** |  |  | **<0.001** |  |  | **<0.001** |
| Q1 | ref. | ref. |  | ref. | ref. |  | ref. | ref. |  |
| Q2 | 1.42 (0.93,2.14) | 0.096 |  | 1.46 (0.96,2.22) | 0.070 |  | 1.23 (0.83,1.82) | 0.290 |  |
| Q3 | 1.78 (1.19,2.65) | **0.004** |  | 1.84 (1.23,2.75) | **0.002** |  | 1.35 (0.91,1.98) | 0.127 |  |
| Q4 | 2.84 (1.94,4.16) | **<0.001** |  | 2.88 (1.96,4.24) | **<0.001** |  | 1.93 (1.32,2.80) | **<0.001** |  |
| **SMuRF-less** | | | | | | | | | |
| **NLR(Tertile)** |  |  | 0.233 |  |  | 0.179 |  |  | 0.221 |
| Q1 | ref. | ref. |  | ref. | ref. |  | ref. | ref. |  |
| Q2 | 0.43 (0.12,1.50) | 0.192 |  | 0.49 (0.13,1.86) | 0.298 |  | 0.49 (0.12,1.94) | 0.315 |  |
| Q3 | 1.10 (0.36,3.30) | 0.862 |  | 1.44 (0.38,5.43) | 0.582 |  | 1.86 (0.46,7.42) | 0.379 |  |
| Q4 | 0.28 (0.03,2.34) | 0.243 |  | 0.34 (0.03,3.02) | 0.336 |  | 0.49 (0.05,4.60) | 0.537 |  |
| **PLR (Tertile)** |  |  | 0.658 |  |  | 0.409 |  |  | 0.452 |
| Q1 | ref. | ref. |  | ref. | ref. |  | ref. | ref. |  |
| Q2 | 1.92 (0.56,6.60) | 0.295 |  | 2.14 (0.59,7.76) | 0.246 |  | 1.81 (0.47,6.88) | 0.379 |  |
| Q3 | 0.70 (0.12,4.00) | 0.697 |  | 0.63 (0.10,3.75) | 0.619 |  | 0.70 (0.11,4.16) | 0.696 |  |
| Q4 | 1.91 (0.30,12.03) | 0.487 |  | 1.64 (0.24,10.94) | 0.604 |  | 1.30 (0.18,9.30) | 0.788 |  |


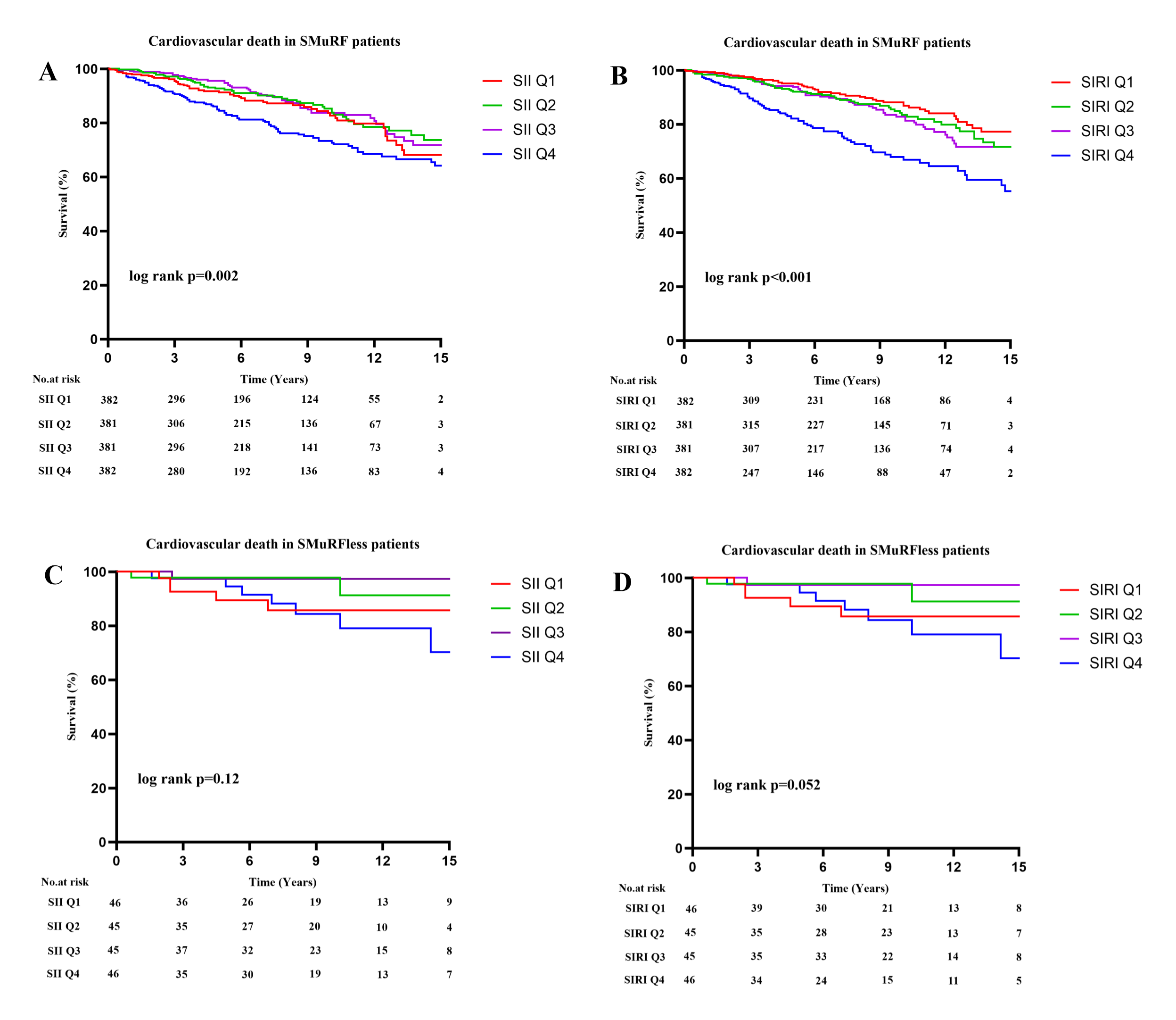


**Supplementary Figure 1 Kaplan-Meier curves for cardiovascular death during follow-up for patients in each subgroup.**

A.Kaplan-Meier curves of patients with SMuRFs grouped according to SII; B.Kaplan-Meier curves of patients with SMuRFs grouped according to SIRI

C.Kaplan-Meier curves of patients with SMuRF-less grouped according to SII; D.Kaplan-Meier curves of patients with SMuRF-less grouped according to SIRI

Abbreviations: SMuRF, standard modifiable risk factors; SII, systemic inflammation index; SIRI, systemic inflammatory response index


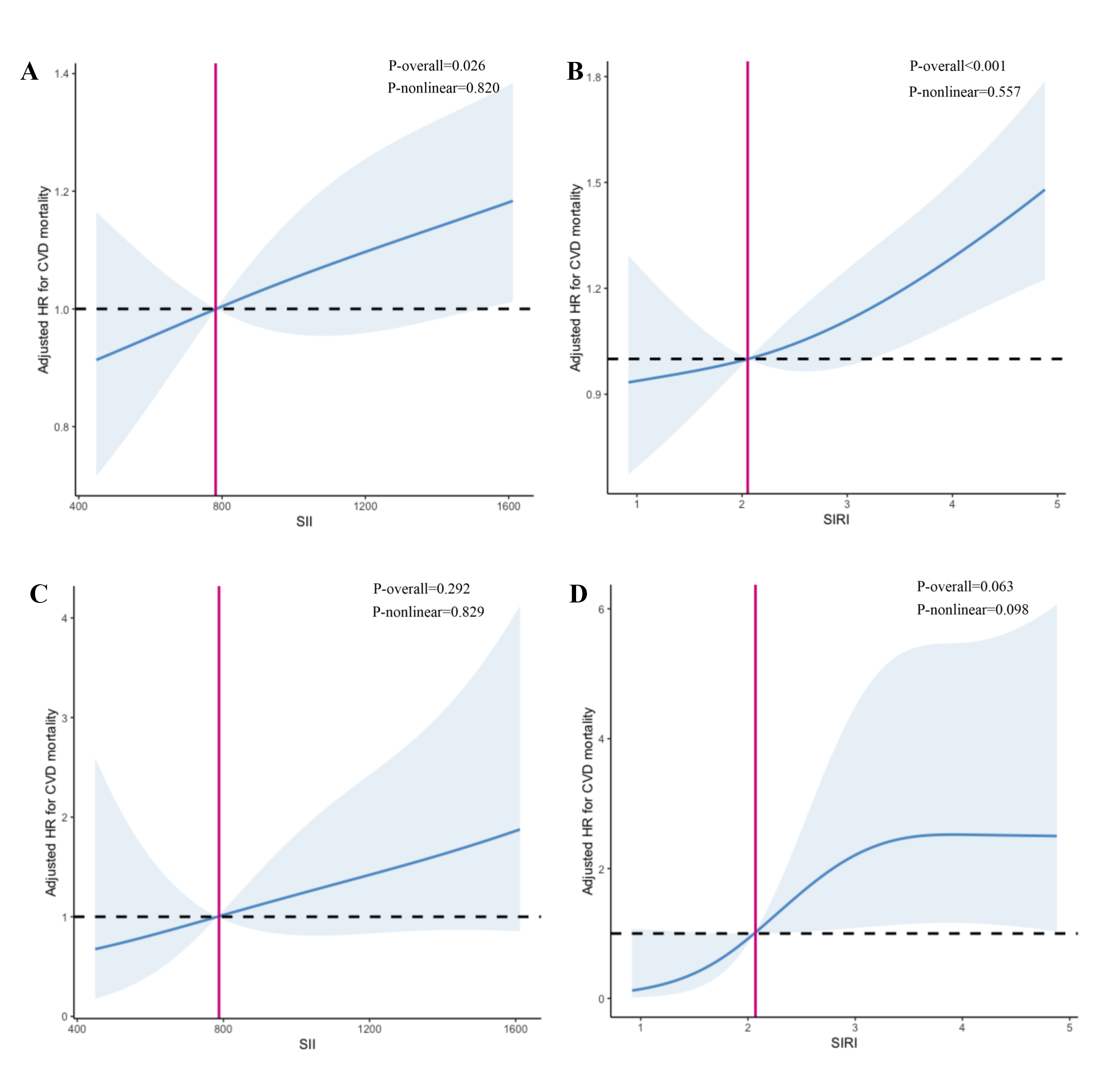


**Supplementary Figure 2 Relationship between SII/SIRI and cardiovascular mortality as assessed by the RCS after correction for covariates.**

A.RCS analysis of patients with SMuRFs grouped according to SII; B.RCS analysis of patients with SMuRFs grouped according to SIRI

C.RCS analysis of patients with SMuRF-less grouped according to SII; D.RCS analysis of patients with SMuRF-less grouped according to SIRI

The solid blue line corresponds to the central estimates and the light blue shading indicates the 95% confidence intervals

Abbreviations: SMuRF, standard modifiable risk factors; SII, systemic inflammation index; SIRI, systemic inflammatory response index; RCS, restricted cubic spline
